# 2-Iodo-3-nitro­pyridine

**DOI:** 10.1107/S1600536809018534

**Published:** 2009-05-29

**Authors:** Li-Hua Mao, Yan Chen

**Affiliations:** a School of City Development, University of Jinan, Jinan 250002, People’s Republic of China; b Shandong Blood Center, Jinan 250014, People’s Republic of China

## Abstract

In the crystal structure of the title compound, C_5_H_3_IN_2_O_2_, inter­molecular C—H⋯N hydrogen-bonding inter­actions link the mol­ecules into one-dimensional chains along the *b* axis.

## Related literature

For the applications of 2-iodo-3-nitro­pyridine in organic synthesis, see: Baik *et al.* (2005 ▶); Choi-Sledeski *et al.* (2003 ▶). For the crystal structure of related compounds, see: Holmes *et al.* (2002 ▶); Saha *et al.* (2006 ▶).
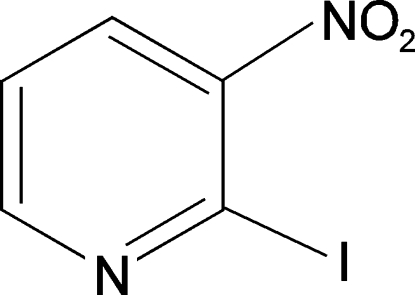


## Experimental

### 

#### Crystal data

C_5_H_3_IN_2_O_2_
*M* *_r_* = 249.99
Monoclinic, 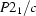

*a* = 8.0169 (15) Å
*b* = 12.313 (2) Å
*c* = 8.0999 (15) Å
β = 119.66 (2)°
*V* = 694.8 (3) Å^3^
*Z* = 4
Mo *K*α radiation
μ = 4.54 mm^−1^
*T* = 298 K
0.60 × 0.30 × 0.21 mm

#### Data collection

Bruker SMART CCD area-detector diffractometer
Absorption correction: multi-scan (*SADABS*; Sheldrick, 1996 ▶) *T*_min_ = 0.147, *T*_max_ = 0.385
3615 measured reflections
1345 independent reflections
1267 reflections with *I* > 2σ(*I*)
*R*_int_ = 0.037

#### Refinement

*R*[*F* ^2^ > 2σ(*F* ^2^)] = 0.030
*wR*(*F* ^2^) = 0.075
*S* = 1.12
1345 reflections
91 parameters
H-atom parameters constrained
Δρ_max_ = 0.50 e Å^−3^
Δρ_min_ = −1.09 e Å^−3^

### 

Data collection: *SMART* (Bruker, 1998 ▶); cell refinement: *SAINT* (Bruker, 1999 ▶); data reduction: *SAINT*; program(s) used to solve structure: *SHELXS97* (Sheldrick, 2008 ▶); program(s) used to refine structure: *SHELXL97* (Sheldrick, 2008 ▶); molecular graphics: *SHELXTL* (Sheldrick, 2008 ▶); software used to prepare material for publication: *SHELXTL*.

## Supplementary Material

Crystal structure: contains datablocks I, global. DOI: 10.1107/S1600536809018534/rz2322sup1.cif

Structure factors: contains datablocks I. DOI: 10.1107/S1600536809018534/rz2322Isup2.hkl

Additional supplementary materials:  crystallographic information; 3D view; checkCIF report

## Figures and Tables

**Table 1:** Hydrogen-bond geometry (Å, °)

*D*—H⋯*A*	*D*—H	H⋯*A*	*D*⋯*A*	*D*—H⋯*A*
C2—H2*A*⋯N2^i^	0.93	2.61	3.529 (5)	172

Symmetry code: (i) 
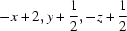
.

**Fig. 1.**
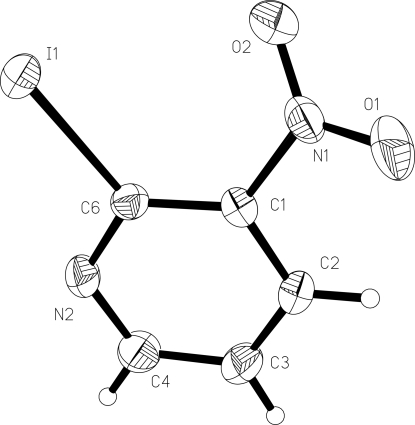
View of the title compound, with displacement ellipsoids drawn at the 40% probability level.

**Fig. 2.**
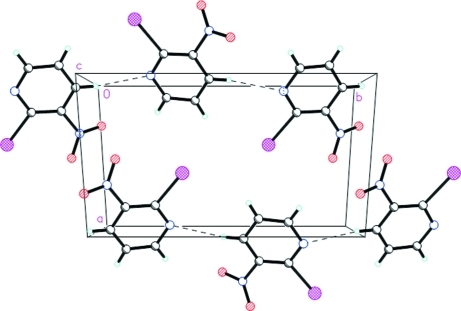
A partial packing diagram of the title compound viewed along the c axis. Intermolecular H bonds are shown as dashed lines.

## supplementary crystallographic information

### Comment

In this paper, we report the crystal structure of the title compound, 2-iodo-3-nitropyridine, which is an important intermediate in organic synthesis (Baik *et al.*, 2005; Choi-Sledeski *et al.*, 2003).

In the molecule of the title compound (Fig. 1), all bond lengths are normal and in a good agreement with those reported previously for 2,6-diiodopyridine (Holmes *et al.*, 2002) and 2-iodo-3hydroxypyridine (Saha *et al.*, 2006). Atoms I1 and N1 are slightly displaced on opposite sides of the pyridine ring by 0.0719 (3) and 0.015 (4) Å, respectively. The nitro group is tilted by 34.6 (3)° with respect to the pyridine ring. The crystal structure is stabilized by intermolecular C—H···N hydrogen bonds (Table 1) linking the molecules into one dimension chains along the *b* axis (Fig. 2).

### Experimental

The title compound was prepared by reaction of 2-amino-3-nitropyridine (1.1 g, 5 mmol), KNO_3_ (1.01 g, 10 mmol), HI (6.6 g, 25 mmol, 50% aqueous solution), CuI (0.48 g, 2.5 mmol) and DMSO (60 ml) at 333K. After neutralizing with an alkaline solution, the reaction mixture was extracted several times with diethyl ether. The combined ethereal extracts were washed with water, dried over anhydrous sodium sulfate and concentrated to afford the crude product. Purification by flash chromatography gave 2-iodo-3-nitropyridine as a yellow solid in 70% isolated yield (0.875 g). Crystals suitable for X-ray diffraction analysis were obtained by slow evaporation of a methanol solution at room temperature over a period of one week.

### Refinement

All H atoms were found on difference maps, with C—H = 0.93 Å and included in the final cycles of refinement using a riding model, with *U*_iso_(H) = 1.2*U*_eq_(C).

### Crystal data

**Table:**

C_5_H_3_IN_2_O_2_	*F*(000) = 464
*M**_r_* = 249.99	*D*_x_ = 2.390 Mg m^−^^3^
Monoclinic, *P*2_1_/*c*	Mo *K*α radiation, λ = 0.71073 Å
Hall symbol: -P 2ybc	Cell parameters from 1077 reflections
*a* = 8.0169 (15) Å	θ = 2.5–25.9°
*b* = 12.313 (2) Å	µ = 4.54 mm^−^^1^
*c* = 8.0999 (15) Å	*T* = 298 K
β = 119.66 (2)°	Block, yellow
*V* = 694.8 (3) Å^3^	0.60 × 0.30 × 0.21 mm
*Z* = 4	

### Data collection

**Table:**

Bruker SMART CCD area-detector diffractometer	1345 independent reflections
Radiation source: fine-focus sealed tube	1267 reflections with *I* > 2σ(*I*)
graphite	*R*_int_ = 0.037
φ and ω scans	θ_max_ = 26.0°, θ_min_ = 2.9°
Absorption correction: multi-scan (*SADABS*; Sheldrick, 1996)	*h* = −9→8
*T*_min_ = 0.147, *T*_max_ = 0.385	*k* = −15→14
3615 measured reflections	*l* = −9→9

### Refinement

**Table:**

Refinement on *F*^2^	Primary atom site location: structure-invariant direct methods
Least-squares matrix: full	Secondary atom site location: difference Fourier map
*R*[*F*^2^ > 2σ(*F*^2^)] = 0.030	Hydrogen site location: inferred from neighbouring sites
*wR*(*F*^2^) = 0.075	H-atom parameters constrained
*S* = 1.12	*w* = 1/[σ^2^(*F*_o_^2^) + (0.0401*P*)^2^ + 0.1824*P*] where *P* = (*F*_o_^2^ + 2*F*_c_^2^)/3
1345 reflections	(Δ/σ)_max_ = 0.001
91 parameters	Δρ_max_ = 0.50 e Å^−^^3^
0 restraints	Δρ_min_ = −1.09 e Å^−^^3^

### Special details

**Table:**

Geometry. All e.s.d.'s (except the e.s.d. in the dihedral angle between two l.s. planes) are estimated using the full covariance matrix. The cell e.s.d.'s are taken into account individually in the estimation of e.s.d.'s in distances, angles and torsion angles; correlations between e.s.d.'s in cell parameters are only used when they are defined by crystal symmetry. An approximate (isotropic) treatment of cell e.s.d.'s is used for estimating e.s.d.'s involving l.s. planes.
Refinement. Refinement of *F*^2^ against ALL reflections. The weighted *R*-factor *wR* and goodness of fit *S* are based on *F*^2^, conventional *R*-factors *R* are based on *F*, with *F* set to zero for negative *F*^2^. The threshold expression of *F*^2^ > σ(*F*^2^) is used only for calculating *R*-factors(gt) *etc*. and is not relevant to the choice of reflections for refinement. *R*-factors based on *F*^2^ are statistically about twice as large as those based on *F*, and *R*- factors based on ALL data will be even larger.

### Fractional atomic coordinates and isotropic or equivalent isotropic displacement parameters (Å^2^)

**Table:**

	*x*	*y*	*z*	*U*_iso_*/*U*_eq_	
I1	0.59372 (3)	0.162020 (19)	0.16542 (4)	0.03968 (14)	
O1	0.7237 (5)	0.5149 (2)	0.3364 (5)	0.0601 (9)	
O2	0.5036 (5)	0.4081 (3)	0.1331 (6)	0.0635 (9)	
N1	0.6704 (5)	0.4373 (3)	0.2292 (5)	0.0395 (7)	
C1	0.8177 (5)	0.3758 (3)	0.2090 (5)	0.0304 (7)	
C2	0.9711 (5)	0.4353 (3)	0.2237 (6)	0.0378 (8)	
H2A	0.9807	0.5095	0.2477	0.045*	
C3	1.1084 (6)	0.3811 (4)	0.2019 (6)	0.0443 (10)	
H3A	1.2127	0.4180	0.2084	0.053*	
C4	1.0884 (5)	0.2712 (4)	0.1703 (6)	0.0465 (11)	
H4A	1.1812	0.2349	0.1544	0.056*	
N2	0.9430 (5)	0.2136 (2)	0.1610 (5)	0.0389 (7)	
C5	0.8074 (5)	0.2654 (3)	0.1774 (5)	0.0300 (7)	

### Atomic displacement parameters (Å^2^)

**Table:**

	*U*^11^	*U*^22^	*U*^33^	*U*^12^	*U*^13^	*U*^23^
I1	0.0391 (2)	0.0313 (2)	0.0496 (2)	−0.00698 (8)	0.02262 (15)	−0.00258 (9)
O1	0.073 (2)	0.0392 (16)	0.074 (2)	0.0116 (15)	0.0408 (19)	−0.0131 (16)
O2	0.0423 (18)	0.0458 (17)	0.107 (3)	0.0055 (15)	0.0404 (19)	0.0006 (19)
N1	0.043 (2)	0.0296 (16)	0.053 (2)	0.0098 (14)	0.0293 (17)	0.0093 (16)
C1	0.0318 (18)	0.0274 (17)	0.0322 (17)	0.0040 (14)	0.0161 (14)	0.0012 (15)
C2	0.040 (2)	0.0275 (17)	0.045 (2)	−0.0054 (15)	0.0204 (18)	−0.0024 (17)
C3	0.038 (2)	0.040 (2)	0.057 (2)	−0.0087 (17)	0.0248 (19)	−0.003 (2)
C4	0.038 (2)	0.043 (2)	0.067 (3)	0.0012 (16)	0.033 (2)	−0.0095 (19)
N2	0.0402 (17)	0.0257 (15)	0.0535 (19)	0.0024 (13)	0.0253 (15)	−0.0042 (15)
C5	0.0270 (16)	0.0306 (18)	0.0291 (16)	−0.0016 (14)	0.0114 (14)	0.0006 (14)

### Geometric parameters (Å, °)

**Table:**

I1—C5	2.097 (3)	C2—H2A	0.9300
O1—N1	1.217 (4)	C3—C4	1.371 (8)
O2—N1	1.222 (5)	C3—H3A	0.9300
N1—C1	1.478 (4)	C4—N2	1.335 (5)
C1—C5	1.378 (5)	C4—H4A	0.9300
C1—C2	1.385 (5)	N2—C5	1.322 (4)
C2—C3	1.372 (6)		
			
O1—N1—O2	124.9 (3)	C4—C3—H3A	120.8
O1—N1—C1	117.6 (3)	C2—C3—H3A	120.8
O2—N1—C1	117.5 (3)	N2—C4—C3	123.6 (3)
C5—C1—C2	120.5 (3)	N2—C4—H4A	118.2
C5—C1—N1	123.2 (3)	C3—C4—H4A	118.2
C2—C1—N1	116.3 (3)	C5—N2—C4	118.5 (3)
C3—C2—C1	117.8 (4)	N2—C5—C1	121.2 (3)
C3—C2—H2A	121.1	N2—C5—I1	113.3 (2)
C1—C2—H2A	121.1	C1—C5—I1	125.4 (2)
C4—C3—C2	118.5 (3)		
			
O1—N1—C1—C5	146.8 (4)	C3—C4—N2—C5	2.2 (6)
O2—N1—C1—C5	−35.1 (5)	C4—N2—C5—C1	−1.9 (5)
O1—N1—C1—C2	−33.1 (5)	C4—N2—C5—I1	−178.8 (3)
O2—N1—C1—C2	145.0 (4)	C2—C1—C5—N2	0.2 (5)
C5—C1—C2—C3	1.3 (6)	N1—C1—C5—N2	−179.7 (3)
N1—C1—C2—C3	−178.7 (4)	C2—C1—C5—I1	176.6 (3)
C1—C2—C3—C4	−1.2 (6)	N1—C1—C5—I1	−3.3 (5)
C2—C3—C4—N2	−0.6 (7)		

### Hydrogen-bond geometry (Å, °)

**Table:**

*D*—H···*A*	*D*—H	H···*A*	*D*···*A*	*D*—H···*A*
C2—H2A···N2^i^	0.93	2.61	3.529 (5)	172

Symmetry codes: (i) −*x*+2, *y*+1/2, −*z*+1/2.
